# Chemical characterization and biological activity data for a novel indirubin derivative, LDD-1819

**DOI:** 10.1016/j.dib.2019.104373

**Published:** 2019-08-09

**Authors:** Woong-Hee Kim, Pyeonghwa Jeong, Seon-Wook Kim, Haaglim Cho, Jeong-min Lee, Shinae Seo, Haihong Shen, Youngkeun Ahn, Da-Woon Jung, Yong-Chul Kim, Darren R. Williams

**Affiliations:** a School of Life Sciences, Gwangju Institute of Science and Technology, 1 Oryong-Dong, Buk-Gu, Gwangju, 61005, Republic of Korea; b Cell Regeneration Research Center, Chonnam National University Hospital, Gwangju, 61469, Republic of Korea

**Keywords:** Metastasis, Cell plasticity, Cellularization, Indirubin derivative, Glycogen synthase kinase-3 β, Aurora kinase A

## Abstract

This article contains chemical characterization and biological activity data for a novel indirubin derivative, termed LDD-1819. The detailed synthesis procedure and associated NMR data are presented. The concentration-dependent inhibition data of two biological targets, glycogen synthase kinase-3β and aurora kinase A are described. The following biological data are also contained in this article: 1) the cellularization of skeletal muscle myotubes by LDD-1819 or two small molecule inhibitors of glycogen synthase kinase-3β and aurora kinase A (BIO and reversine) and gene expression data for the myoblast markers Pax-7 and Myf5, 2) Cell viability of hTERT human immortalized fibroblasts, colon cancer cells and breast cancer cells, and 3) Western blotting analysis of full length and cleaved caspse-7, and cleaved poly (ADP-ribose) polymerase (PARP) in hTERT fibroblasts treated with LDD-1819. A schematic diagram of the biological activities of LDD-1819 is also presented. Further interpretation and discussion of these data are provided in the associated research article ‘A novel indirubin derivative that increases somatic cell plasticity and inhibits tumorigenicity’ (Kim et al., 2019).

**Table:** Specifications Table

Subject	Chemistry (General)
Specific subject area	Bioorganic and Medical Chemistry
Type of data	Images Graphs Figures
How data were acquired	NMR spectroscopy (JNM-ECX400P, JEOL), microscopy (Olympus's CLX41), western blot imaging system (LAS500, AB Biosciences), microplate reader (VersaMax, Molecular Devices).
Data format	Analyzed.
Parameters for data collection	Synthesis and cell plasticity/anti-cancer activity after treatment with a novel indirubin derivative, LDD-1819.
Description of data collection	The parameters were as follows: 1) ^1^H NMR spectra of the chemical structure of compound LDD-1819. 2) Cell viability after LDD-1819 treatment. 3) Microscopic observation of myotube cellularization. 4) Quantitative PCR analysis of gene expression related to myogenesis, apoptosis or cancer cell invasion. 5) Inhibition activity against glycogen synthase kinase-3 β and aurora kinase A.
Data source location	Gwangju Republic of Korea (South Korea).
Data accessibility	With the article
Related research article	Author's name: Kim WH, Jeong P, Kim SW, Cho H, Lee JM, Seo S, Shen H, Ahn Y, Jung DW, Kim YC, Williams DR. Title: A novel indirubin derivative that increases somatic cell plasticity and inhibits tumorigenicity Journal: Bioorganic & Medicinal Chemistry. https://doi.org/10.1016/j.bmc.2019.05.025

**Table:** 

**Value of the data** • This data describes the chemical characterization of novel indirubin derivative, LDD-1819, which modulates cell plasticity and inhibits tumorigenicity. • This data provides further characterization of the biological activity of compound LDD-1819. • Overall, this data should allow the scientific community to ascertain the potential utility of LDD-1819 for development as an anti-cancer agent or chemical tool to facilitate cell reprogramming protocols.

## 1 Data

**Fig. 1:**
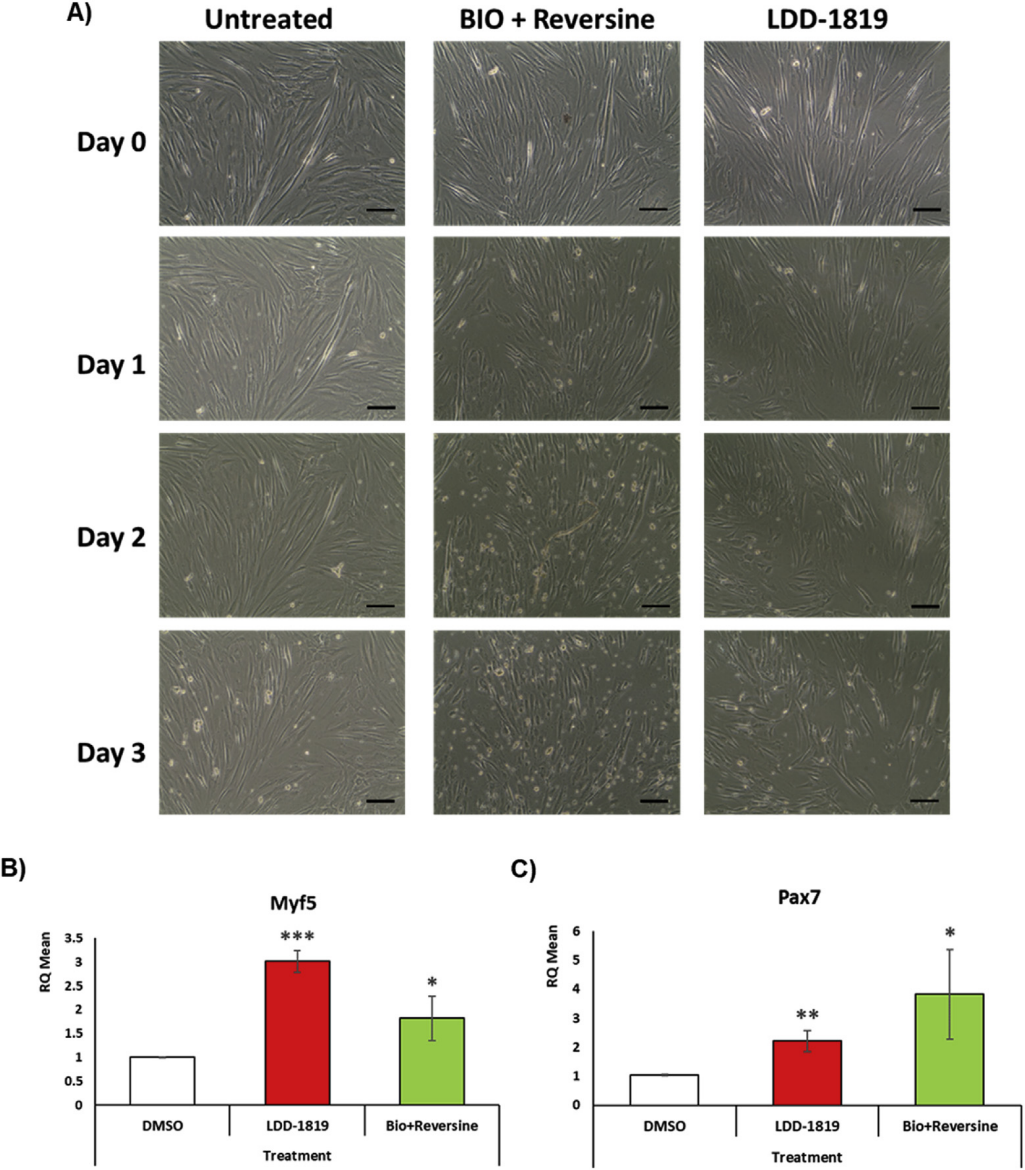
(A) Microscopic indication of cellularization in myotubes treated with 1 μM LDD-1819, or 5 μM BIO and 100 nM reversine, for 72 h in differentiation media. Scale bar = 100 μm. (B) qPCR analysis of the expression of myoblast marker genes Myf5 and Pax7, which are down-regulated during differentiation, in myotubes treated with 5 μM BIO and 100 nM reversine, or 1 μM LDD-1819, for 72 h. *, **, or**** = *p* < 0.05, *p* < 0.01 or *p* < 0.001 compared to myotubes treated with 0.1% DMSO. RQ = mean expression level for the gene of interest.

C2C12 myotube cultures were treated with LDD-1819, or the compounds BIO and reversine (a glycogen synthase kinase-3 β (GSK-3β) inhibitor and an aurora kinase A inhibitor) [2]. Using microscopic analysis, evidence of cellularization in the myotubes could be observed after treatment with LDD-1819 or BIO plus reversine (Fig. 1A). qPCR analysis was carried out for Pax-7 and Myf5 expression, which are genes expressed in undifferentiated myoblasts and downregulated during differentiation into multinucleated myotubes [[3], [4]]. Treatment with LDD-1819, or BIO plus reversine, increased Pax-7 and Myf5 expression, which supports the observation of cellularization and reversal of the multinucleated myotube differentiated state (Fig. 1B and C).

**Fig. 2:**
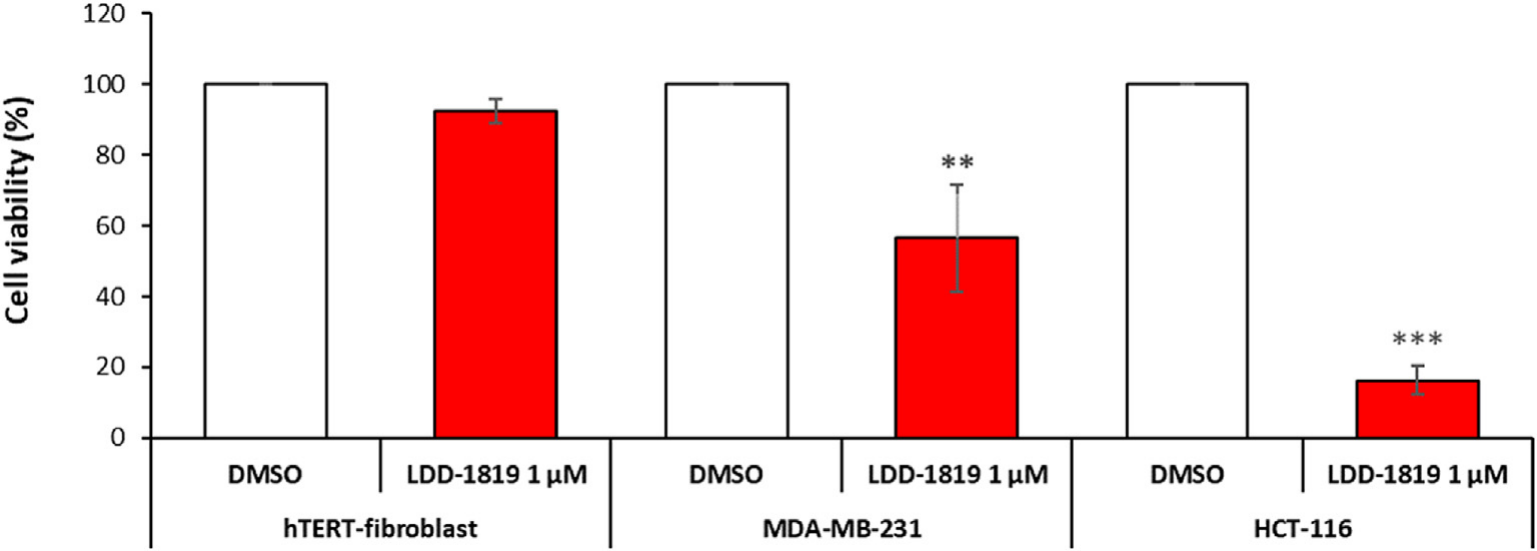
MTT assay to measure cell viability in hTERT immortalized human fibroblasts (non-cancer cells), MDA-MB-231 human breast cancer cells and HCT-116 human colon cancer cells treated with LDD-1819 for 72 h. ** = *p* < 0.01 and *** = *p* < 0.001 compared to 0.1% DMSO treated cells.

**Fig. 3:**
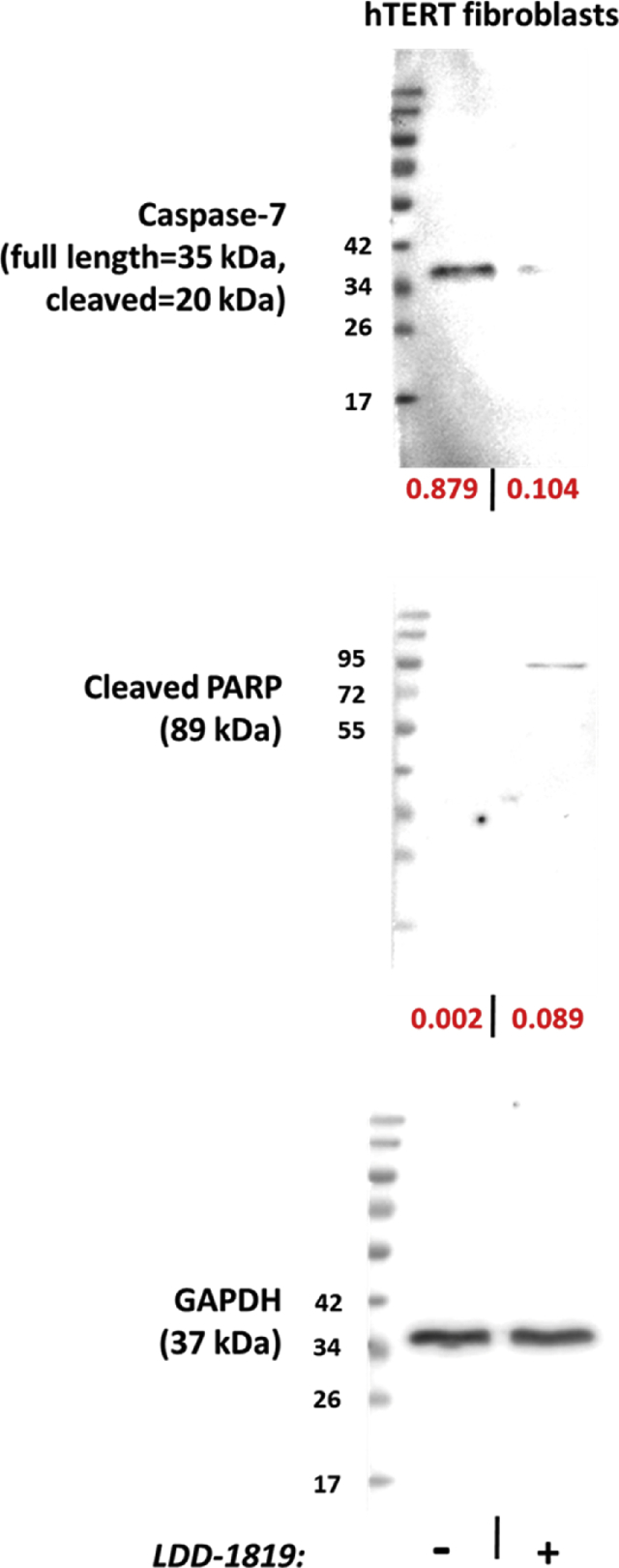
Western blotting analysis of full length or cleaved caspase-7, and PARP expression, in hTERT fibroblasts treated with 500 nM LDD-1819 for 72 h. GAPDH was used as a housekeeping gene control. Fold-change in protein expression is shown as red numbers beneath each panel.

**Fig. 4:**
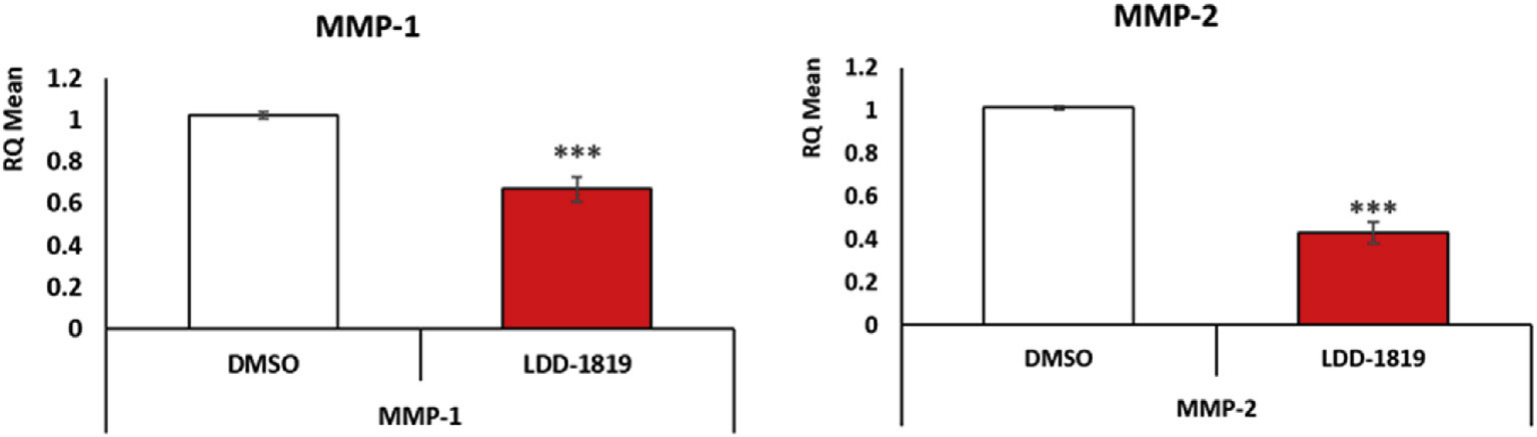
qPCR analysis of MMP-1 and MMP-2 expression in MDA-MB-231 breast cancer cells treated with 50 nM LDD-1819 for 24 h. *** = *p* < 0.001 compared to 0.1% DMSO treated cells.

The anti-cancer activity was investigated in human, normal hTERT immortalized fibroblasts, and human breast and colon carcinoma cells (MDA-MB-231 and HCT116 cells). Cell viability data showed that LDD-1819 treatment was selectively cytotoxic for the cancer cells, with a greater toxic response in the colon carcinoma cells compared to the breast carcinoma cells (Fig. 2). The expression level of the pro-apoptosis proteins cleaved caspase-7 and cleaved Poly (ADP-ribose) polymerase (PARP) was measured in untreated and LDD-1819 treated hTERT fibroblasts. It was observed that LDD-1819 treatment did not produce detectable levels of cleaved caspase-7 and reduced the expression of full length cleaved caspase-7 (Fig. 3). Cleaved PARP was detected in hTERT fibroblasts after treatment with LDD-1819 (Fig. 3.). The expression level of two enzymes linked to cancer cell invasion (matrix metalloproteinase (MMP)-1 and MMP-2) was measured in LDD-1819 treated breast carcinoma cells using qPCR. LDD-1819 treatment reduced the expression of MMP-1 and MMP-2 in the cancer cells (Fig. 4).

**Fig. 5:**
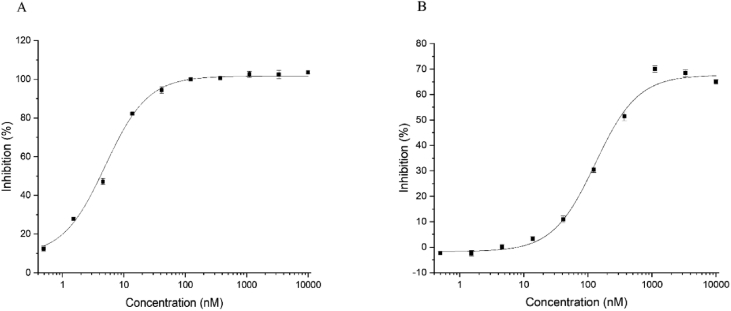
Concentration dependent inhibition of GSK-3β (A) and aurora kinase A (B) by LDD-1819. IC_50_ for GSK-3β = 5.46 nM; IC_50_ for aurora kinase A inhibition = 222 nM.

**Fig. 6:**
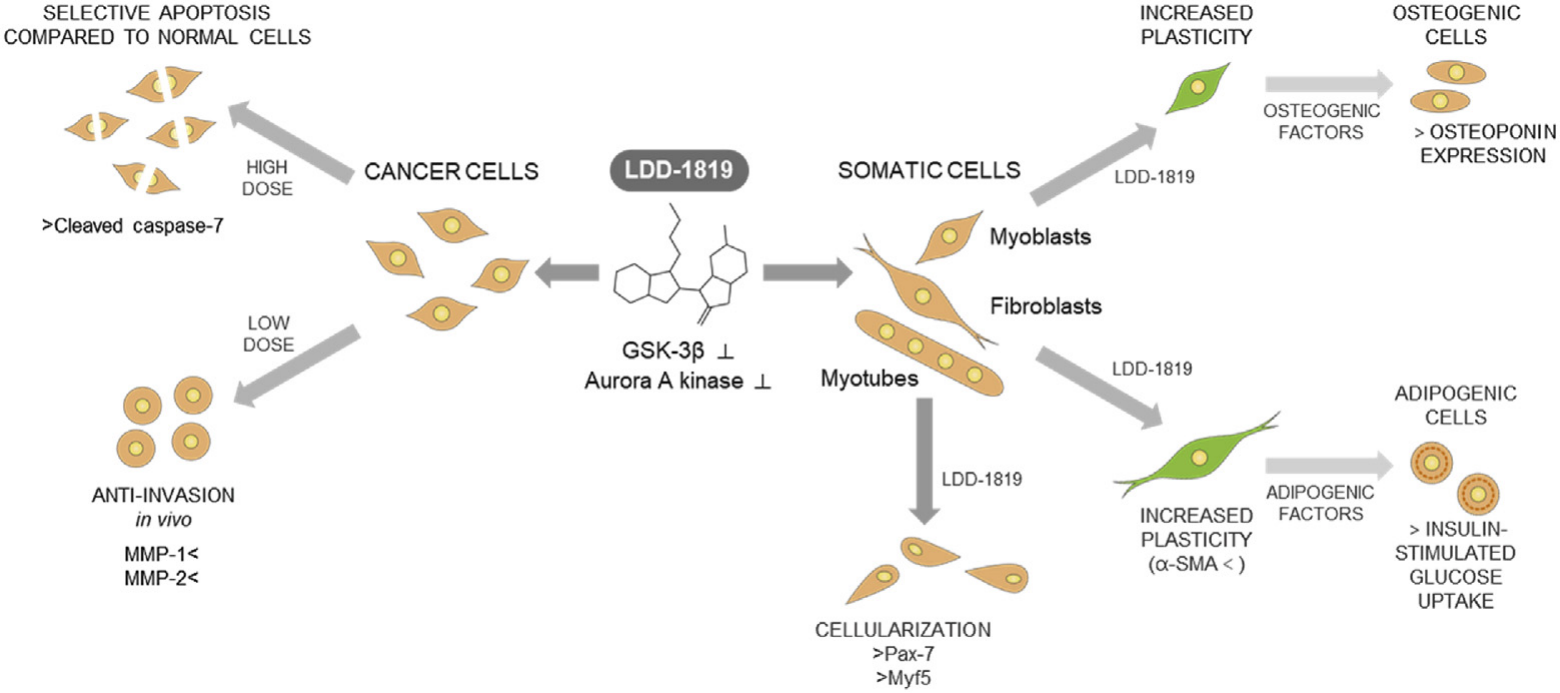
Schematic diagram of the biological effects of compound LDD-1819 in cancer cells and somatic cells.

The inhibitory activity of LDD-1819 against two biological targets, GSK-3β and aurora kinase A, was measured in a concentration dependent experiment (Fig. 5). The IC_50_ was calculated as 5.46 nM for GSK-3β and 222 nM for aurora kinase A. A schematic diagram summarizing the biological effects of compound LDD-1819, based on the data herein and in the associated research article ‘A novel indirubin derivative that increases somatic cell plasticity and inhibits tumorigenicity’ [1] is shown in Fig. 6.

## 2 Experimental design, materials, and methods

### 2.1 Reagents and antibodies

BIO ((2′Z,3′E)-6-Bromoindirubin-3′-oxime), CelLytic™ M lysis buffer, (β-D-arabinofuranosyl) cytosine) (AraC), and TRI-Solution™ were purchased from Sigma-Aldrich. Reversine was purchased from Santa Cruz Biotechnology, Inc. An antibody for PARP (catalogue number #9541) was purchased from Cell Signaling Technology. An antibody recognizing both full length and cleaved caspase-7 (catalogue number #9492) was also purchased from Cell Signaling Technology. An antibody for GAPDH was purchased from Santa Cruz Biotechnology, Inc (catalogue number sc-365062).

### 2.2 Synthetic procedure of LDD-1819

To a solution of 5-hydroxyanthranilic acid (1) in 50 mL of methanol was added 0.5 mL of acetic acid, followed by ethyl glyoxalate and NaCNBH3. The reaction mixture was stirred for overnight at room temperature. Then the methanol was removed by evaporation. The residue was taken up in a solution of saturated NH4Cl in water and extracted with ethyl acetate (3 times). The combined extracts were dried over sodium sulfate, filtered and evaporated. The residue was purified by silica gel column chromatography (CHCl3:CH3OH = 30:1) to give the ester derivative (2) which was subsequently hydrolyzed in 1N NaOH(aq) and methanol. The reaction mixture was stirred for 1 hour at room temperature and the resulting solution was neutralized by 1N HCl to afford the solid product (3) which was filtered and washed with water. To the di-acid compound (3) was added acetic anhydride and Na2CO3. The reaction mixture was refluxed for 4 hours and the product was extracted with ethyl acetate (3 times) and washed with water. The combined extracts were dried over sodium sulfate, filtered and evaporated. The residue was purified by silica gel column chromatography (hexane: ethyl acetate = 2:1) to give diacetate compound (4). Indirubin oxime compound, 6 was synthesized from aldol condensation of the indoxyl-N,O-diacetate (4) with 5-nitroisatin under basic conditions to yield the compound (5), followed by the reaction with hydroxylamine in pyridine with reflux condition for 6 hour. The reaction mixture was acidified by 1N HCl, filtered and washed with water to obtain N-oxime compound (6) [5]. Next, a solution of 2-(Boc-amino)ethyl bromide, N-oxime analog (6) and potassium carbonate in DMF was stirred at ambient temperature for overnight. The resulting mixture was partitioned in saturated aq. NH4Cl solution and ethyl acetate (3 times). The organic phase was evaporated and the product (7) was purified by silica gel column chromatography. Finally, to a solution of compound 7 in THF, 4N HCl in 1,4-dioxane was added dropwise in ice-bath. After the completion of reaction, the precipitate was filtered and washed with DCM. The resulting product was dried in vacuum oven to afford the hydrochloride salt, LDD-1819.

### 2.3 Analytical data of the compound

Starting materials, reagents, and solvents were purchased from Aldrich Chemical Co. (Milwaukee, WI) and TCI (Tokyo) and used as supplied without further purification. Thin-layer chromatography (TLC) was performed on fluorescent silica gel plates (60 F_254_ from Merck) and visualized with either short-wave UV light. Chromatographic purifications were achieved using kieselgel 60 (Merck) 0.040–0.063 mm column chromatography. ^1^H NMR spectra were determined with a JEOL JNM-LA 300WB spectrometer at 300 MHz or JEOL JNM-ECX 400P spectrometer at 400 MHz, and spectra were taken in CDCl_3_ or DMSO-*d*_6_ or D_2_O. Unless otherwise noted, chemical shifts are expressed as ppm downfield from internal tetramethylsilane, or relative ppm from DMSO (2.5 ppm), H_2_O (4.79 ppm).

#### 2.3.1 2-((2-ethoxy-2-oxoethyl)amino)-5-hydroxybenzoic acid (2)

Yield: 89%, ^1^H NMR ^1^H NMR (400 MHz, DMSO-*d*_*6*_) δ ppm 8.72 (s, 1H), 7.22 (s, 1H), 6.86-6.83 (dd, *J* = 8.8, 2.8 Hz, 1H), 6.46-6.43 (d, *J* = 8.8 Hz, 1H), 4.14-4.09 (q, *J* = 7.2 Hz, 2H), 3.98 (s, 2H), 1.20-1.16 (t, *J* = 6.8 Hz, 3H).

#### 2.3.2 2-((carboxymethyl)amino)-5-hydroxybenzoic acid (3)

Yield: 90%, ^1^H NMR ^1^H NMR (400 MHz, DMSO-*d*_*6*_) δ ppm 8.73 (s, 1H), 7.22 (s, 1H), 6.86-6.83 (dd, *J* = 8.8, 2.8 Hz, 1H), 6.44-6.41 (d, *J* = 8.8 Hz, 1H), 3.89 (s, 2H).

#### 2.3.3 1-acetyl-1H-indole-3,5-diyl diacetate (4)

Yield: 32%, ^1^H NMR (300 MHz, CDCl_3_) δ ppm 8.36 (d, *J* = 9.3 Hz, 1H), 7.95(s, 1H), 7.31 (d, *J* = 2.4 Hz, 1H), 7.15-7.12(dd, *J* = 9.3, 2.4 Hz, 1H), 2.67(s, 3H), 2.36 (s, 3H), 2.31(s, 3H). ESI [M+H] = 214.16.

#### 2.3.4 (Z)-5-hydroxy-5′-nitro-[2,3′-biindolinylidene]-2′,3-dione (5)

Yield: 32%, ^1^H NMR (300 MHz, DMSO-*d*_*6*_) δ ppm 11.78 (s, 1H), 11.51 (s, 1H), 9.59 (d, J = 2.4 Hz, 1H), 8.27-8.25 (d, J = 9.16 Hz, 1H), 8.14-8.11 (dd, J = 8.84, 2.44 Hz, 1H), 7.52-7.48 (m, 2H), 7.12-7.07 (m, 2H)

#### 2.3.5 (2Z,3E)-5-hydroxy-3-(hydroxyimino)-5′-nitro-[2,3′-biindolinylidene]-2′-one (6)

Yield: 66%, ^1^H NMR (400 MHz, DMSO-*d*_*6*_) δ ppm 13.87 (s, 1H), 11.78 (s, 1H), 11.35 (s, 1H), 9.41 (d, *J* = 2.8 Hz, 1H), 9.32 (s, 1H), 8.05 (dd, *J* = 11.6, 2.8 Hz, 1H), 7.76 (d, *J* = 3.2 Hz, 1H), 7.29 (d, *J* = 11.6 Hz, 1H), 7.04 (d, *J* = 11.2 Hz, 1H) 6.86 (dd, *J* = 11.2, 3.2 Hz, 1H).

**Figure:**
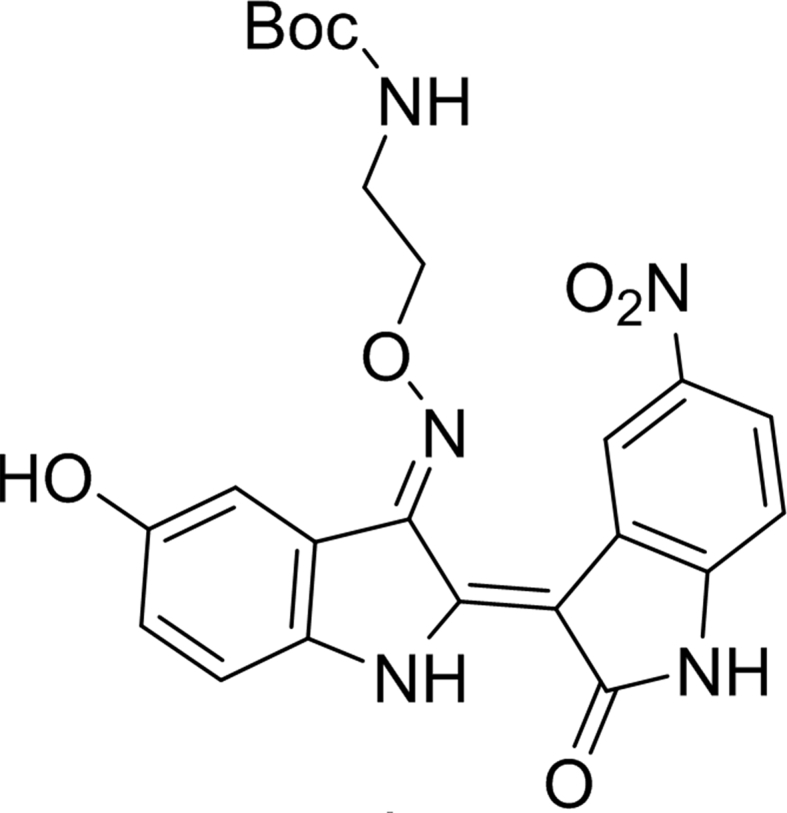

#### 2.3.6 *tert*-butyl (2-((((2Z,3E)-5-hydroxy-5′-nitro-2′-oxo-[2,3′-biindolinylidene]-3-ylidene)amino)oxy)ethyl)carbamate (7)

Yield: 48%, 1H NMR(400 MHz, DMSO-d6) δ ppm 11.66 (s, 1H), 11.39 (s, 1H), 9.85 (s, 1H), 9.36 (s, 1H), 8.10-8.07 (dd, *J* = 8.8, 2.4 Hz, 1H), 7.65 (d, *J* = 2.4 Hz, 1H), 7.13–7.29 (d, *J* = 8.8 Hz, 1H), 7.07-7.03 (m, 2H), 6.92-6.89 (dd, *J* = 8.8, 2.4 Hz, 1H), 4.71-4.69 (t, *J* = 5.6 Hz, 2H), 3.55-3.54 (m, 2H), 1.31-1.24 (brs, 9H).

HRMS (FAB) m/z calculated for C_23_H_23_N_5_O_7_ [M+H]^+^ 482.16, found 482.1679.

**Figure:**
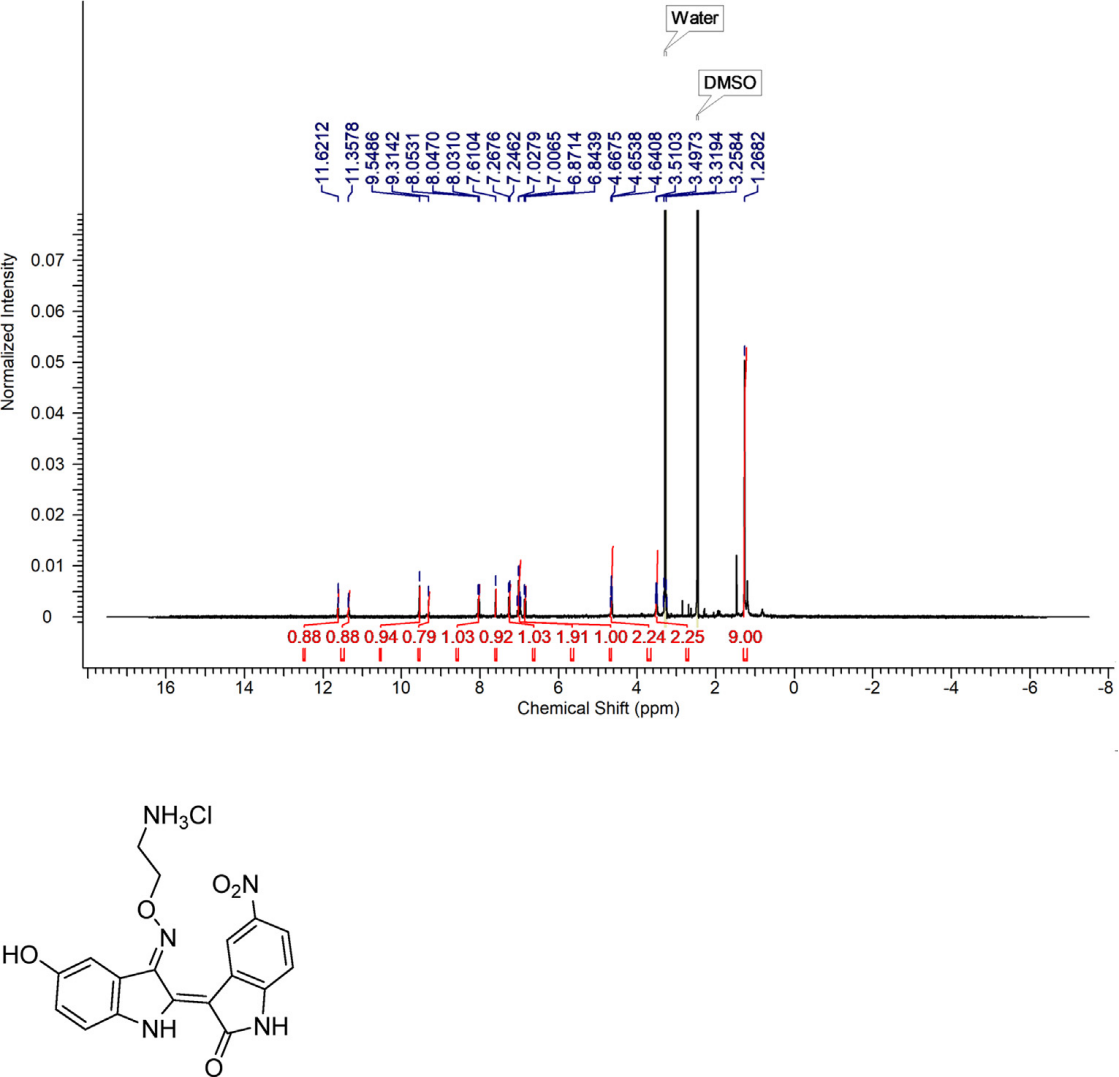

#### 2.3.7 (2Z,3E)-3-((2-aminoethoxy)imino)-5-hydroxy-5′-nitro-[2,3′-biindolinylidene]-2′-one hydrochloride (LDD-1819)

Yield: 98%, 1H NMR(400 MHz, DMSO-d6) δ ppm 11.66(s, 1H), 11.47(s, 1H), 9.51(d, J = 2.4 Hz, 1H), 9.48(s, 1H), 8.24(brs, 3H), 8.09(dd, J = 8.4, 2.4 Hz, 1H), 7.78(d, J = 2.4 Hz, 1H), 7.30 (d, J = 8.4 Hz, 1H), 7.07 (d, J = 8.8 Hz, 1H), 6.95 (dd, J = 8.4, 2.4 Hz, 1H), 4.87 (t, J = 4.4 Hz, 2H), 3.49 (t, J = 4.4 Hz, 2H).

HRMS (FAB) m/z calculated for C_18_H_15_N_5_O5_7_ [M-Cl]^+^ 381.11, found 382.1152.

**Figure:**
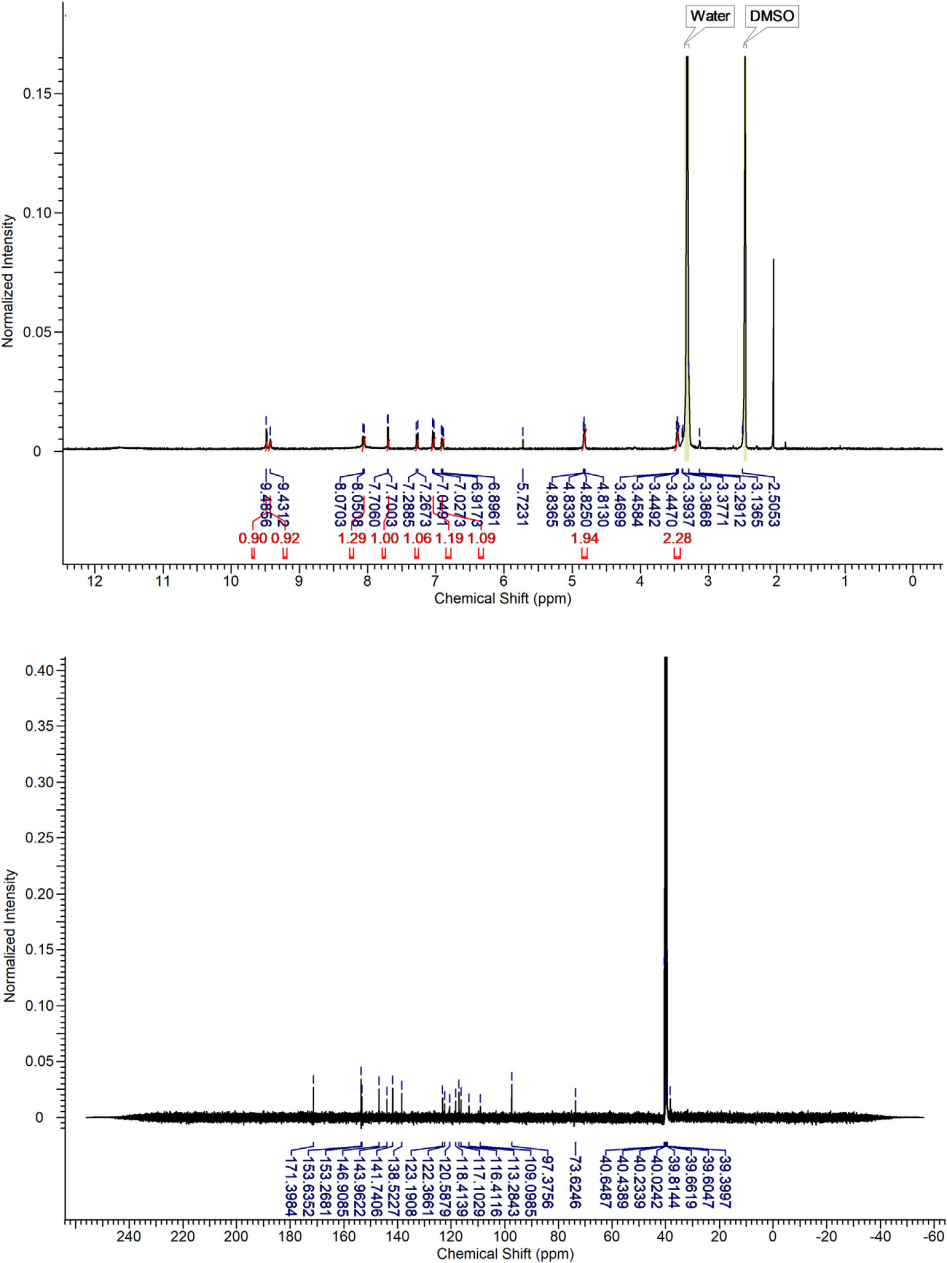

### 2.4 Cell culture

C2C12 murine skeletal myoblasts, HCT116 human colon cancer cells and MDA-MB-231 human breast cancer cells were cultured in Dulbecco's modified Eagle's medium (DMEM) containing 10% fetal bovine serum (FBS), 100 units/mL penicillin and 100 μg/mL streptomycin (PenStrep). Confluent cultures of C2C12 myoblasts were induced to undergo differentiation into myotubes by incubation with differentiation media (DMEM containing 2% horse serum and PenStrep) for 5 d. The differentiation media was replenished every 24 h. To remove undifferentiated myoblasts, the cultures were treated with 50 μM AraC for 3 d (2d after the onset of differentiation), using the previously published protocol [6].

hTERT expressing immortalized human fibroblasts (hTERT fibroblasts) were a generous gift from Professor Jin Kim, Department of Oral Pathology, Yonsei University, Republic of Korea. hTERT fibroblasts were cultured with F-media (DMEM + F-12 media at a 3:1 ratio) supplemented with 10% FBS and 1% PenStrep.

### 2.5 Cell toxicity assay

Cytotoxicity was measured using the 3-(4,5-dimethylthiazol-2-yl)-2,5-diphenyltetrazolium bromide, a tetrazole (MTT) assay, as previously described [7] using a VersaMax plate reader (Molecular Devices). Cell viability was assessed after 72 h compound treatment.

### 2.6 Western blotting analysis

Proteins were separated using 10% SDS-PAGE and transferred onto 0.2 μm nitrocellulose (Bio-Rad). Densitometry analysis of band intensity was carried out with Image J 1.54s software (National Institutes of Health, USA).

### 2.7 Quantitative PCR (qPCR) analysis of Pax7, Myf5, MMP-1 and MMP-2 expression

RNA was harvested using the TRIzol reagent (Thermo Fisher Scientific, MA, USA) following the manufacturer's instructions. For qPCR, the transcript level of the gene of interest was measured using the StepOnePlus Real Time PCR System (Applied Biosystems, UK). Total RNA was reverse transcribed into cDNA using the AccuPower® RT PreMix (Bioneer Corporation) and used for real-time PCR in accordance with the manufacturer's guidelines, and PCR was performed in triplicate in a total volume of 20 μL TOPrealTM qPCR 2X PreMIX (SYBR Green with high ROX) (Enzynomics co. Ltd., South Korea) containing 250 nM final concentration of the specific primer and 1 μL cDNA. The mixture was incubated for 10 min at 95 °C prior to PCR amplification, which consisted of 40 cycles of denaturation, annealing and extension. Denaturation was carried out at 15 s at 95 °C, annealing was carried out at 1 min at 60 °C and extension was carried out at 72 °C for 20 s with fluorescence detection at 72 °C after each cycle. When the final cycle was completed, melting-point analysis was performed within the range of 60–95 °C and continuous fluorescence detection. A specific cDNA sample was included in each run and used as a reference for comparison between runs. The final mRNA expression in each experiment was calculated as the average value of three independent sets of experiments. The primer sequence for murine Myf5 was AGCTGGGCAGAATACGTGCTT (forward) and AGAACAGGCAGAGGAGAATCCA (reverse; product size 112 b.p.) and the primer sequence for murine Pax7 was CACAGAGGCAGAGCTGATTGC (forward) and CCAATTGAGGAGAGTGACAGGTT (reverse; product size 157 b.p.). The primer sequence for mouse GAPDH was CTCCACTCACGGCAAATTCA (forward) and GCCTCACCCCATTTGATGTT (reverse; product size 120 b.p.) The primer sequence for human MMP-1 was GGACCTGGAGGAAATCTTGCT (forward) and TCCAAGAGAATGGCCGAGTT (reverse; product size 150 b.p.) and the primer sequence for human MMP-2 was CGTCTGTCCCAGGATGACATC (forward) and CTGTTTGCAGATCTCAGGAGTGA (reverse; product size 120 b.p.). The primer sequence for human GAPDH was AAGGTCGGAGTCAACGGATTT (forward) and TTGACGGTGCCATGGAATTT (reverse; product size 170 b.p.).

### 2.8 Western blotting analysis

Proteins were separated by 10% SDS-PAGE and transferred onto 0.2 μm nitrocellulose (Bio-Rad). Densitometry analysis of band intensity was carried out using the Image J 1.54s software (National Institutes of Health, USA).

### 2.9 Measurement of aurora kinase A inhibition

Inhibitory activity against Aurora kinase A was measured using Z′-LYTE® Biochemical assay based on the differential sensitivity of phosphorylated and non-phosphorylated peptides to proteolytic cleavage. The inhibitory activity of compounds was evaluated on the ATP (Km value), Aurora kinase A (0.91 - 10 ng) and Synthetic FRET peptide (2 μM) concentration. The kinase reaction buffer consists of 50 mM HEPES pH 7.5, 0.01% BRIJ-35, 10 mM MgCl2, 1 mM EGTA. The total reaction volume was 10 μL and serial dilutions of compounds, FRET peptide and ATP were incubated. Kinase reactions were performed for 30 min at room temperature in standard 384-well plates, and then 5 μL of the development reagent solution was treated to the reaction plates 1 h before reading the plates. After reagents were added for detection, the multi-label reader was set at 445 nm for excitation, and 520 nm for emission. The FRET emission ratio was represented as ratio F445/F520 nm. The IC_50_ values were calculated with nonlinear regression analysis using OriginPro 9.1 software (OriginLab, Northampton, MA).

### 2.10 Measurement of GSK-3β kinase inhibition

Inhibitory activity against GSK-3β was measured using Z′-LYTE® Biochemical assay based on the differential sensitivity of phosphorylated and non-phosphorylated peptides to proteolytic cleavage. The inhibitory activity of compounds was evaluated on the ATP (Km value), GSK-3β (0.22 - 0.92 ng) and Synthetic FRET peptide (2 μM) concentration. The kinase reaction buffer consists of 50 mM HEPES pH 7.5, 0.01% BRIJ-35, 10 mM MgCl2, 1 mM EGTA. The total reaction volume was 10 μL and serial dilutions of compounds, FRET peptide and ATP were incubated. Kinase reactions were performed for 30 min at room temperature in standard 384-well plates, and then 5 μL of the Development Reagent solution was treated to the reaction plates 1 h before reading the plates. After reagents were added for detection, the multi-label reader was set at 445 nm for excitation, and 520 nm for emission. The FRET emission ratio was represented as ratio F445/F520 nm. The IC_50_ values were calculated with nonlinear regression analysis using OriginPro 9.1 software (OriginLab, Northampton, MA).

### 2.11 Statistical analysis

Statistical significance was determined using the Student's t-test (Microsoft Excel, 2016). A *p* value < 0.05 was deemed to be significant. Unless otherwise stated, all data shown are representative of three experimental repeats and the error bars for the graphs are standard deviation*.*
